# The Impact of PRRS Eradication Program on the Production Parameters of the Hungarian Swine Sector

**DOI:** 10.3390/ani13091565

**Published:** 2023-05-07

**Authors:** István Szabó, Imre Nemes, László Búza, Ferenc Polyák, Ádám Bálint, Gábor Fitos, Derald J. Holtkamp, László Ózsvári

**Affiliations:** 1 National PRRS Eradication Committee, H-1021 Budapest, Hungary; nemes.imre@mstsz.eu; 2 Intervet Hungaria Ltd., H-1095 Budapest, Hungary; laszlo.buza@yahoo.com; 3 Tedej Agricultural Production and Service Co., H-4085 Hajdúnánás, Hungary; polyakferenc@tedejrt.hu; 4 National Food Chain Safety Office Veterinary Diagnostic Directorate, H-1143 Budapest, Hungary; 5 Hungarian Association for Porcine Health Management, Hungary, H-2053 Herceghalom, Hungary; fitos.gabor@mstsz.eu; 6 Department of Veterinary Diagnostic and Production Animal Medicine, College of Veterinary Medicine, Iowa State University, Ames, IA 50011, USA; holtkamp@iastate.edu; 7 Department of Veterinary Forensics and Economics, University of Veterinary Medicine, H-1078 Budapest, Hungary; ozsvari.laszlo@univet.hu; 8 National Laboratory of Infectious Animal Diseases, Antimicrobial Resistance, Veterinary Public Health and Food Chain Safety, University of Veterinary Medicine Budapest, H-1078 Budapest, Hungary

**Keywords:** PRRS, eradication program, production parameters

## Abstract

**Simple Summary:**

The results of our study demonstrate that the PRRS eradication program applying the herd depopulation–repopulation approach led to a considerable improvement of the productivity of the Hungarian pig herd. This result also demonstrates that, independently of the PRRS eradication, it is still necessary to consider investments in the individual production units to increase efficiency, and to carry out herd depopulation–repopulation in cases where the current genetics limits improvements in productivity.

**Abstract:**

Background: The Hungarian national eradication program of PRRS was successfully completed between 2014 and 2022. There were doubts about the efficiency of the eradication program in Hungary from the beginning to the tune that it might only be carried out efficiently through depopulation–repopulation of the infected herds, which is a very costly procedure. In our study, we investigated the impact of the depopulation–repopulation procedure, which played a prominent role in the PRRS eradication program on the productivity of the Hungarian swine sector–namely, on the number of slaughter pigs per sow per year and the total live slaughter weight per sow per year. Material and Methods: Since 2014, we monitored the evolution of the PRRS eradication through the depopulation–repopulation approach on the large-scale breeding herds in Hungary. Most producers replaced their herds with animals that were free of PRRS and other infectious diseases (mycoplasmosis, actinobacillosis, swine dysentery, atrophic rhinitis, etc.). On this basis, we evaluated the change in the number of slaughter pigs per sow per year as a consequence of depopulation–repopulation of the herds being carried out. In the statistical analysis linear regression was used. Conclusions: The results of our study demonstrate that the PRRS eradication program with the herd depopulation–repopulation approach led to a considerable improvement of the productivity of Hungarian pig farming. This result also demonstrates that, independent of the PRRS eradication, it is still necessary to consider investments into the individual production units to increase efficiency, and to carry out herd depopulation–repopulation in cases where the current genetics limits improvements in productivity.

## 1. Introduction

The importance of the pig sector in Hungary is reflected by the fact that in 2018, it accounted for 8.2% (HUF 222 billion ≈ €550 million) of the total gross output of the agricultural sector (HUF 2720 billion ≈ €6.8 million), while it represented 24% of the total output of animal products [1]. The sector has undergone significant changes in recent decades. Between 2000 and 2019, the pig population decreased by one-fifth for backyard farmers and by 13% for commercial entities. During that same period, the sow population decreased by one-fifth for backyard farmers and by 38% for commercial entities. The pig sector had infectious diseases such as Aujeszky disease and porcine reproductive and respiratory syndrome (PRRS), which caused significant economic losses. After finishing Aujeszky disease eradication in 2006 [2], Hungary implemented a national eradication program of PRRS in 2014. Porcine reproductive and respiratory syndrome (PRRS) is currently one of the most damaging infectious diseases in the swine industry worldwide [3,4,5,6,7].

The majority of PRRS cases were caused by the European type of the virus, but the American type PRRS virus also appeared (mainly of vaccine origin). At the beginning of the program cc. 4% of the backyard farms [8], 63.9% of the large-scale fattening farms were PRRS positive [9], respectively. In 2014, out of 470 large-scale breeding units, 125 were infected [10]. A total of 85% of the large-scale swine farms in Hungary are farrow-to-finish type.

In Hungary, the national eradication program of PRRS was successfully completed between 2014 and 2022 [10]. The national PRRS eradication program in Hungary was based on a territorial principle, and it was obligatory for each swine farm. One of the main objectives of the PRRS eradication program was to increase the competitiveness of the Hungarian pig industry by:

- Eliminating losses due to PRRS.
- Decreasing antibiotic usage level during production.
- Increasing the profitability of producers.

PRRS eradication on the large-scale swine farms was carried out mainly by the depopulation–repopulation method [10,11]. It was a major issue of the eradication program from the beginning that it might only be carried out efficiently through depopulation–repopulation of the infected herds, which is a very costly procedure. At the same time, this is the process that allows the utilization of the newest, most productive genetics in the swine sector.

In large-scale commercial farrow-to-finish pig farms, the number of slaughter pigs per sow per year is arguably the most important indicator of profitability on a swine farm from a financial point of view because it is strongly associated with revenue and the efficient use of inputs. The countries that have been at the forefront of pig farming have already reached and even exceeded 32 slaughter pigs per sow per year [12].

There are no official data about this parameter regarding the Hungarian pig industry. In 2005, Nyárs [13] stated that the average number of weaned piglets per sow per year was 15.8 in Hungary, which was lower than it was 20 years ago, and which was an unacceptably poor performance by today’s standards. According to Bartha [14], in 2008, the average number of slaughter pigs per sow per year was 16.8 in Hungary, while 22.7 was regarded as good result, and 30 as outstanding. Nagy and Aliczki [15] concluded in 2014 that one of the most significant disadvantages of the Hungarian pig farmers compared to their main competitors was that the number of weaned piglets per sow per year was only 16.8 according to the official statistical data. Based on their data, even the annual number of slaughter pigs/sows could only be significantly lower.

We studied the impact of the depopulation–repopulation procedure, which plays a prominent role in the PRRS eradication program:

- In a certain swine farm, which allows us to conduct the financial analysis of the depopulation –repopulation procedure on the farm level.
- On the productivity of the Hungarian swine sector––namely, on the number of slaughter pigs per sow per year and the total live slaughter weight per sow per year.

## 2. Materials and Methods

### 2.1. Estimation of the Production and Financial Impact of the Depopulation–Repopulation in a Large Farrow-to-Finish Swine Farm

In the first part of our study, we analyzed an individual case in which a swine farm implemented the PRRS eradication program by the depopulation–repopulation process. This farrow-to-finish swine farm had 1400 sows and their progeny and the herd became infected with the PRRS virus in 2003. After the infection, the herd was continuously vaccinated against PRRS virus by using modified-live and/or inactivated PRRS vaccines, applying different vaccination protocols. The modified-live vaccine used exclusively was Porcilis PRRS (MSD). Laboratory monitoring tests were carried out regularly to determine the spread of PRRS virus within the herd. Based on the test results, it seemed that piglets could be raised virus-free for a longer period (7–8 months) until the end of the pre-fattening period, but reinfection occurred from time to time on the farm. By 2020, the progress of the national PRRS eradication program forced the farm to complete its eradication process, and the failure of the previous vaccination methods convinced the farm management that the only viable way to eradicate the PRRS virus would be the depopulation–repopulation approach.

Depopulation–repopulation began in this herd with the cessation of inseminations and was completed with the first slaughter pig sales. The whole period lasted 588 days. The main steps of the transition between the start and finish dates were as follows. After insemination stopped, the continuous sales of breeding sows and fatteners started in a specific order. To speed up the depopulation process, even piglets weighing up to 20–30 kg were sold. After the pens were emptied, a complete external and internal clean-up, including physical cleaning, washing, and disinfection were carried out. Afterwards cleaning and disinfection of the manure lagoon, ceiling, technological equipment, water- and wastewater pipes, manure storage, and removal of contaminated soil from the area around the buildings (10,000 m^2^, 10 cm width, total 1000 m^3^) took place, which was followed by a 2-month resting period (to break-up of the infection chain) when the pig farm was completely empty. In the meantime, the necessary biosecurity investments were carried out (e.g., renovation and enlargement of the social premises, relocation of the fence, animal ramp, etc.), and all pens and their surroundings were disinfected again at the same time.

The repopulation was carried out by purchasing and stocking breeding gilts and the necessary number of boars over a period of 1 month (altogether 1600 breeding animals). The isolation (quarantine) of the animals with the same health status was carried out in the disinfected farm (besides the balanced repopulation of the breeding facilities), which is why it was important for the breeding gilts to come from the same swine herd. This was followed by the onset of the inseminations and the repopulation of sow pens with individual stalls and afterwards group stalls. The start of farrowing then took place, followed by weaning and piglet rearing. The final phase was the full repopulation of the fattening pens and the sales of the first slaughter pigs. Attention was paid to changes in the demand for personnel. Maintenance and tractor staff was hired permanently, but cc. 50% of the caretakers were not hired for 10 months after depopulation. The remaining caretakers were permanently employed during the cleaning and disinfection of the farm. In the analysis, we should also take into account the changes in feed demand. If the feed supply, or at least a part of it, comes from the plant growing, its sales can reduce the costs of depopulation–repopulation.

Based on the financial data of the swine farm of Tedej Agricultural Production and Service Co., we analyzed the costs of the eradication through depopulation–repopulation and the change in annual income resulting from the expected improvement in the production parameters of the farm.

We also assessed the length of the payback period for the necessary investment, which included the inevitable loss in production and revenue after the depopulation–repopulation.

### 2.2. Estimation of the Economic Impact of the Depopulation–Repopulation Procedure on the Entire Swine Sector in Hungary

In the second part of our study, in order to assess the economic impact of the depo-pulation–repopulation procedure on the entire swine sector in Hungary, we estimated the impact of the PRRS eradication program on one of the most important productivity parameters in pig farming—the average number of slaughter pigs per sow per year. This parameter is not registered in the Hungarian official statistics. In order to calculate the average number of slaughter pigs per sow per year, the following public statistical databases were used:

- The total number of pigs, including sows, in Hungary as of 1st of December, according to the Hungarian Central Statistical Office [16].
- Data available in the Agricultural Statistics Information System (ASIR) of the Institute of Agricultural Economics (AKI), which shows that from 2012 onwards, the total number of pigs slaughtered, including the culled sows, their average live slaughter weight, and the average carcass weight. The total number of slaughter pigs is the margin between the total number of pigs slaughtered and the total number of slaughtered (culled) sows in Hungary [17].
- The FELIR system of the National Food Chain Safety Office (NÉBIH) and the EU Sante Traces system give information about the annual number of imported live pigs for immediate slaughter and the annual number of exported live pigs for immediate slaughter from Hungary [18].

**Figure 1:**
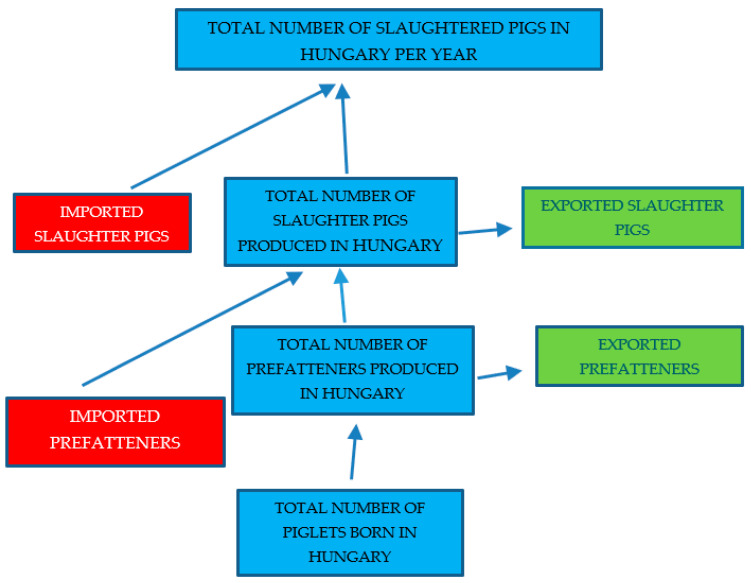
The calculation method of the number of slaughter pigs produced per sow per year in Hungary.

Figure 1 shows the way of calculation to have the total number of slaughter pigs being produced annually in Hungary as follows: the total number of slaughter pigs produced per sow per year = ((total number of igs slaughtered per year—total slaughtered (culled) sows per year)—total number imported live pigs for immediate slaughter—total number of imported prefatteners + total number exported prefatteners + total number of exported live pigs for immediate slaughter)/total number of sows per year.

The impact of the progress of the PRRS eradication was analyzed in terms of the change in the annual slaughter pig production per sow, with 2014 as the base year. While the first year of the national PRRS eradication program in Hungary was 2014, the depopulation–repopulation approach started to play a significant role in the process only in 2015. The PRRS eradication program started on 1 January 2014. At the same time, the Hungarian government reduced the VAT rate on live pigs from 27% to 5% in order to reduce the prevalence of illegal sales (tax evasion). Based on the above, we analyzed the annual performance of the swine sector under the same conditions over a period between 2014 and 2022. We performed a linear regression analysis to evaluate the association between the live-weight produced pig per sow per year [kg/sow/year] and then the number of slaughtered pigs [head/sow/year] and the years. Statistical analyses were performed in R version 4.1.2. [16,17,18]. The level of significance was set to 0.05.

Since 2014, we monitored the evolution of the PRRS eradication through the depopulation–repopulation approach on the large-scale breeding herds in Hungary. It is important to emphasize that producers electing to depopulate and repopulate to eradicate PRRS were entitled to get state compensation, which was the margin between the breeding value of the sows and their slaughter value (unless there was a reason for not compensating the farmers, e.g., biosecurity or disease control deficiencies). This compensation was conditional on restocking the farm after its depopulation and disinfection with the same number of animals depopulated. On this basis, we evaluated the change in the number of slaughter pigs per sow per year as a consequence of depopulation–repopulation of the herds carried out.

## 3. Results

### 3.1. Estimated Production and Financial Impact of the Depopulation–Repopulation in a Large Farrow-to-Finish Swine Farm

The impact of the depopulation–repopulation on the production and financial parameters in the 1400-sow, farrow-to-finish swine farm analyzed is shown in Table 1.

According to the calculations, the imposed operation-related costs were paid back within one year of operation in a fully populated pig farm operating at full capacity. This means that by the end of the 3rd year after the start of the depopulation of the farm, the full cost of the depopulation–repopulation approach will be paid back.

### 3.2. Estimated Economic Impact of the Depopulation–Repopulation Procedure on the Entire Swine Sector in Hungary

In order to analyze the results of the PRRS eradication program at national level, it was necessary to estimate the annual number of slaughtered (culled) sows due to the era-dication since 2014 in the large-scale swine farms using the depopulation–repopulation approach. The data in Table 2 show that 9.2% of the total number of sows culled between 2014 and 2022 were sent to slaughterhouses because of the PRRS eradication. Larger values were found in 2019, 2020, and 2021, which were the final years of the PRRS eradication program. It is important to emphasize that in 2014, in the year of the reduction of VAT on live animals from 27% to 5%, there was no significant impact on this indicator.

The impact of the progress of the national PRRS eradication program on the total slaughter pig production per sow per year was compared to 2014 as a base year (Table 3). It can be seen that after 2014, the improvement in the total number of slaughter pigs per sow per year increased in line with the progress of depopulation–repopulation in the swine farms. In 2015, the parameter was still the same as in 2014, but in 2016 and 2022, it was 10% and 32% higher, respectively. This is also reflected in the fact that in 2022, the Hungarian swine sector produced slightly (−2.5%) fewer slaughter pigs with significantly (−26.2%) fewer sows than in 2014.

The trend of changes is quite similar for the live slaughter weight production per sow per year (Table 4). It can be stated that after the reduction of the VAT in 2014, the improvement in the live slaughter weight production per sow per year was in harmony with the progress of depopulation–repopulation approach in the farms eradicating PRRS. Although in 2015, this parameter slightly decreased temporarily compared to the previous year, in 2016 and 2022 it was 13% and 37% higher, respectively. In 2022, the total live slaughter weight production increased by 0.5% compared to 2014, despite the fact that 26.2% less sows were kept in Hungary.

**Figure 2:**
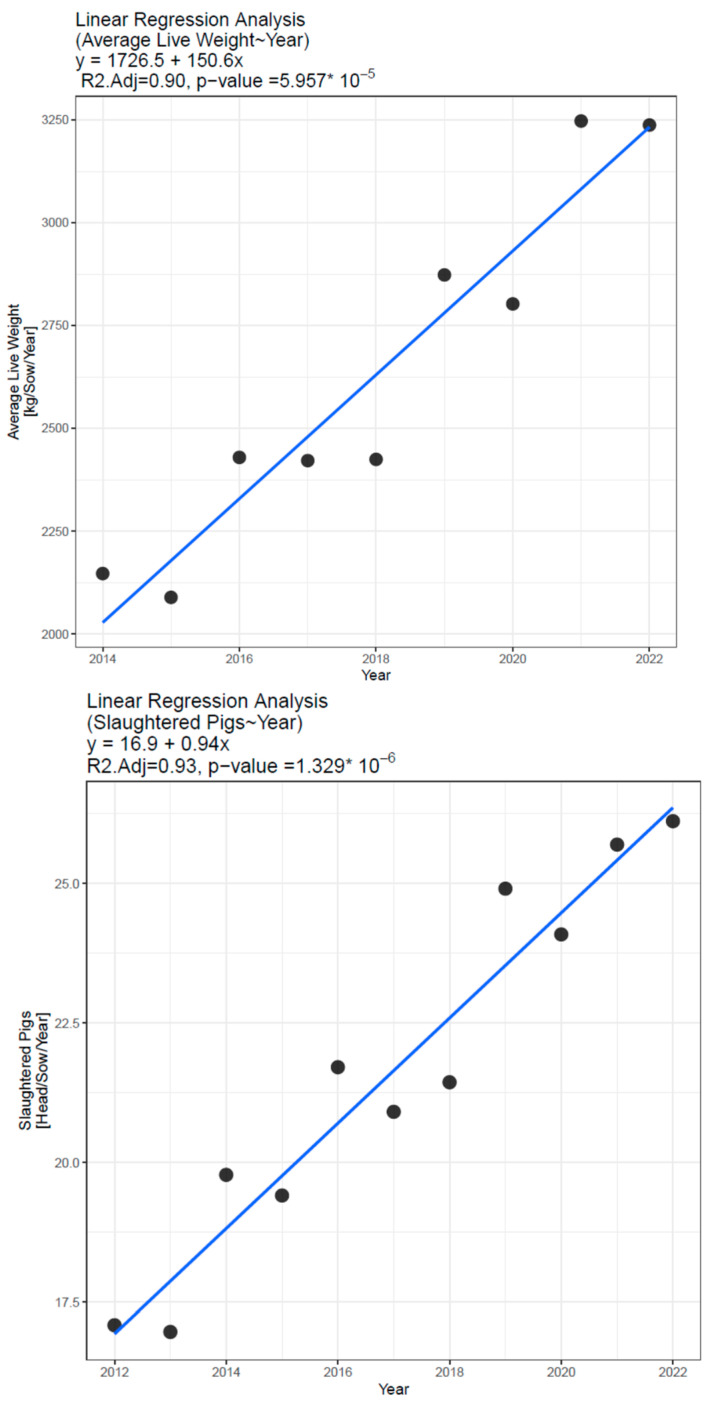
Linear regression analysis of the association between the year and the slaughtered pigs[head]/sow/year and the average live weight of slaughtered pigs[kg/sow/year].

We observed a statistically significant association between the year and the average live weight of slaughtered pigs per sow and the number of slaughtered pigs per sow (*p* < 0.001, in both cases). The adjusted R-squared values were higher than 90% in both cases, indicating a very strong association between the variables. Based on the linear regression model, the live weight of slaughtered pigs increased by 150.6 kg/sow/year, and the number of slaughtered pigs increased by 0.94 pigs/sow/year (Figure 2).

Table 5 shows the financial implications of the better performance of the entire Hungarian swine sector. The annual income per sow increased by 30% in EUR in 2021 compared to 2014, the starting year of the PRRS eradication program (no data are available yet for 2022).

It is also worth highlighting that the depopulation–repopulation approach in the case of the presented large-scale, farrow-to-finish swine farm resulted in a significant increase, from 21 to 25, in the total number of slaughtered pigs per sow per year between 2014 and 2021. In the same period, this production parameter grew from 19.8 to 25.7 slaughter pigs per sow per year in the entire Hungarian pig population (and reached 26.1 in 2022).

## 4. Discussion

In Hungary, the national eradication program of PRRS was successfully completed between 2014 and 2022 [10]. In the EU Member States with a traditionally developed pig population, the prevalence of PRRS in the swine herds is extremely high. There are only four countries where, according to the OIE data, there were no cases (in Norway and Finland) or where there were just sporadic cases (in Sweden in 2007, and in Switzerland in 2012, 2013, 2014 and 2020), so their swine herds can be regarded as PRRS-free. The Danish PRRS eradication has been taking place for years [21].

During the national PRRS eradication program in the Hungarian, large-scale, swine herds, the depopulation–repopulation was the most commonly used method to achieve a free status. This approach provided the farms the opportunity to upgrade their biosecurity measures and to get repopulated with animals having the most modern genetics and the highest available animal health status. Therefore, the PRRS eradication program, starting in 2014 and resulting in Hungary declaring freedom from the disease in 2022, has led to a significant improvement in the profitability of the swine sector. Based on our calculations, the most decisive production parameter in Hungary was the total number of slaughtered pigs per sow per year being only 17.1 in 2012, but it reached 26.1 by 2022, equaling the European average (0.94 slaughter pigs/sow/year increase annually in the defined period).

According to Hoste, the production performance of the European swine farms, expressed as the number of slaughter pigs produced per sow per year, steadily increased since 2010 [12]. However, the increase varied from country to country, and Germany, Denmark, and Belgium led this improvement, with an annual increase of about 0.6 slaughter pigs per sow per year since 2006. Between 2010 and 2018, this production parameter increased from 26 to around 32 in Denmark, from 22 to 25 in Spain, and from over 23 to above 28 in Germany, respectively. In Ireland, 26.8 pigs were produced per sow per year in 2019 [22]. In addition to the number of slaughter pigs per sow per year index, the average carcass weight of slaughter pigs per sow per year can also be a decisive production parameter on the swine farms. In the United States of America, the average carcass weight of slaughter pigs produced per sow per year was about 398.3 kg (878 lb) per sow in 1930, compared to 1905 kg (4200 lb) in 2015, which means an almost 2% per yearly increase [23]. In China, 1943 kg of carcass per sow per year were produced in 2012, while in 2018, 2319 kg of carcass weight per sow per year were produced [24]. In Hungary, the carcass weight per sow per year was not measured, but the strongly correlated live weight of the slaughtered pigs per sow per year index increased by 150.6 kg annually between 2014 and 2022, reaching 3238 kg by the end of that period. 

The profitability of the swine sector is influenced by the total number of slaughter pigs per sow year and their total live weight per sow per year. In a 500-sow pig farm, either increasing from 26.9 to 27.9 or decreasing from 26.9 to 25.9, the number of slaughter pigs per sow per year will result in a 37,000 EUR change in the farm profit, considering an average slaughter live weight of 112 kg [3]. Based on these results, it can be concluded that the 147,800 sows in Hungary generated almost 122.2 million EUR (≈43.9 billion HUF) more income in 2022 than in 2014, the starting year of the eradication.

It can also be stated that under field conditions in Hungary in the late 2010s, the costs of a depopulation–repopulation process of a large-scale swine farm were paid back within one year of operation in a fully populated pig farm operating at full capacity. This means that by the end of the third year after the start of the depopulation of the farm, the full cost of the depopulation–repopulation approach will be paid back. This process, which was forced during national PRRS eradication in Hungary, helped individual farms get the newest genetics, renew worn-out equipment, and rethink the internal and external biosecurity of the farm.

## 5. Conclusions

The results of our studies demonstrate that as a direct consequence of the national PRRS eradication program under Hungarian field conditions, in which applying the herd depopulation–repopulation approach was the main process to reach PRRS free status of the swine herds, led to a considerable improvement of the productivity of the Hungarian pig industry at both individual farm and at country level.

## Figures and Tables

**Table 1:** The changes in the production parameters after depopulation/repopulation in a farrow-to finish, large-scale swine herd with 1400 sows in Hungary.

Parameters		2020 (before Depopulation)	2022 (after Repopulation)	Difference
**Average number of live piglets per litter**		11.0	12.5	+1.5
Preweaning mortality (%)		7	8	+1
Weaned piglets per litter		10.2	11.5	+1.3
Litters/sow/year		2.36	2.40	+0.04
Number of weaned piglets per sow per year		24	27.6	+3.6
Average sow number per farm		1400	1420	+20
Total number of weaned piglets per farm		33,600	39,192	+5592
ADG during nursery (g/day)		425	440	15
FCR during nursery		1.7	1.5	−0.2
ADG during fattening (g/day)		750	900	+150
FCR during fattening		3.0	2.7	−0.3
Average number of days till slaughter		175	155	−20
Average number of slaughter pigs per sow per year		21	25	+4
Average live weight at slaughter (kg)		109	110	+1
Total weight of slaughtered pigs per farm (t)		3205	3905	+700
Total cost of vaccinations and curative treatments per farm (ths HUF)		104,000	54,000	−50,000
Total cost of vaccinations and curative treatments per farm (ths EUR) *		290	150	−139
Total feed consumption per farm per year (t)		10,500	11,000	+500
Annual income (ths HUF)		1,291,194	1,613,205	+322,011
Annual income (ths EUR) *		3596	4493	+897
**Costs**	**n**	**Unit price (HUF)**	**Total cost (HUF)**	**Total cost (€)**
Gilts	1600	100,000	160,000,000	445,620
Boars	5	500,000	2,500,000	6963
Biosecurity investments			30,000,000	83,554
Decrease in income in the transition period			71,381,329	198,806
*State compensation for PRRS eradication*			*−110,932,720*	*−308,962*
Total investment costs			152,948,609	425,981
**Payback time (year)**				**0.48**
**Benefit/cost ratio**				**2.11**

* Average exchange rate in 2021: 1 € = 359.05 HUF.

**Table 2:** Total number of slaughtered sows and the number of slaughtered sows due to PRRS eradication in Hungary (2012–2022).

Year	Slaughtered Sows Due to PRRS Eradication	Slaughtered Sows Due to PRRS Eradication (%)	Total Number of Slaughtered Sows *	Slaughtered Sows Compared to 2012 (%)	Slaughtered Sows Due to PRRS Eradication/Total Slaughtered Sows (%)
2012	0	0%	77,317	100	0.00
2013	0	0%	86,401	112	0.00
2014	0	0%	71,353	92	0.00
2015	3318	5%	96,847	125	3.43
2016	6340	9%	105,703	137	6.00
2017	7860	11%	83,614	108	9.40
2018	4973	7%	92,950	120	5.35
2019	12,494	18%	86,434	112	14.45
2020	7790	11%	75,545	98	10.31
2021	25,697	38%	91,084	118	28.21
2022	2300	3%	66,032	85	3.48
**TOTAL**	**70,772**		**769,562**		**9.20**

* AKI ASIR, 2023.

**Table 3:** Total number of slaughtered pigs in Hungary and the number of slaughtered pigs/sow/year (2012–2022).

Year	Total Slaughtered Pigs (Ths Head)	Change (2012 = 100%)	Total Slaugh-Tered Sows (Head)	Total Slaugh-Tered Pigs (Head)	Imported Prefat-Teners (Head)	Imported Slaughter Pigs (Head)	Exported Prefat-Teners (Head)	Exported Slaughter Pigs (Head)	Total Exported Prefat-Teners and Slaughter Pigs (Head)	Slaughter Pigs Produced in Hungary (Head)	# of Sows (‘000 Heads)	# of Slaugh-Tered Pigs/ Sow/ Year	Change (2012 = 100%)	Change (2014 = 100%) *
2012	3,836,044	100%	77,317	3,758,727	126,812	675,293	9220	456,800	466,020	3,422,642	200.3	17.09	**100%**	86%
2013	3,749,825	98%	86,401	3,663,424	255,925	668,774	2230	482,255	484,485	3,223,210	189.9	16.97	99%	86%
2014	4,077,531	106%	71,353	4,006,178	212,857	389,131	283,947	271,150	555,097	3,959,287	200.2	19.78	116%	**100%**
2015	4,458,502	116%	96,847	4,361,655	517,112	442,609	217,784	199,188	416,972	3,818,906	196.8	19.41	114%	98%
2016	4,675,646	122%	105,703	4,569,943	765,384	414,392	252,439	209,420	461,859	3,852,026	177.4	21.71	127%	110%
2017	4,755,692	124%	83,614	4,672,078	989,560	517,012	279,295	141,802	421,097	3,586,603	171.5	20.91	122%	106%
2018	4,704,599	123%	92,950	4,611,649	750,291	498,917	185,992	265,530	451,522	3,813,963	177.9	21.44	125%	108%
2019	4,620,023	120%	86,434	4,533,589	742,968	466,156	244,416	299,106	543,522	3,867,987	155.3	24.91	146%	126%
2020	4,701,729	123%	75,545	4,626,184	592,127	558,929	161,595	303,778	465,373	3,940,501	163.6	24.09	141%	122%
2021	4,868,985	127%	88,661	4,780,324	679,571	528,799	202,822	257,631	460,453	4,032,407	156.9	25.70	150%	130%
2022	4,532,370	118%	66,032	4,466,338	634,469	485,593	294,336	220,004	514,340	3,860,616	147.8	26.12	153%	132%
	**AKI ASIR Database**			**Calculated Value**	**NÉBIH ENAR Database**					**Calculated Value**	**KSH Database**	**Calculated Value**		

Note: * means ‘VAT’ year, # means number.

**Table 4:** Total live weight of slaughtered pigs in Hungary and the total live weight of slaughtered pigs/sow/year (2014–2022).

Year	Total Slaughter Weight Production (t)	Change (2014 = 100%)	Total Live Weight of Slaughtered Sows (t)	Total Live Weight Slaughtered Pigs (t)	Total Live Weight of Imported Prefatteners * (t)	Total Live Weight of Imported Slaughter Pigs (t)	Total Live Weight of Exported Prefatteners * (t)	Total Live Weight of Exported Slaughter Pigs (t)	Total Live Weight of Slaughtered Pigs Produced in Hungary (t)	# of Sows (‘000 Heads)	Change (2014 = 100%)	Total Live Weight of Slaughtered Pigs (kg/sow/year)	Change (2014 = 100%)
2014	455,935	100%	15,295	440,640	6386	42,801	8518	29,824	429,796	200.2	100%	2146.8	100%
2015	463,948	102%	19,046	444,902	15,513	45,147	6534	20,318	411,092	196.8	98%	2088.9	97%
2016	488,324	107%	21,023	467,301	22,962	42,374	7573	21,414	430,954	177.4	89%	2429.3	113%
2017	491,039	108%	16,325	474,714	29,687	52,532	8379	14,408	415,282	171.5	86%	2421.5	113%
2018	490,840	108%	18,692	472,149	22,509	51,080	5580	27,185	431,325	177.9	89%	2424.5	113%
2019	495,546	109%	16,749	478,797	22,289	49,231	7332	31,589	446,198	155.3	78%	2873.1	134%
2020	514,147	113%	15,208	498,939	17,764	60,281	4848	32,763	458,505	163.6	82%	2802.6	131%
2021	575,017	126%	19,690	555,327	20,387	61,430	6085	29,929	509,523	156.9	78%	3247.4	151%
2022	531,562	115%	14,778	516,784	19,034	51,261	8831	23,224	478,544	147.8	74%	3237.7	151%
	**AKI ASIR Database**			**Calculated Value**	**NÉBIH ENÁR Database**				**Calculated Value**	**KSH Database**		**Calculated Value**	

* 30 kg/head, # means number.

**Table 5:** Change in income of slaughter pigs per sow per year at 2014 price level (2014 vs. 2021).

Year	Slaughter Pigs per Sow per Year	Average Live Weight per Slaughter Pig (kg)	Average Market Price of Slaughter Pigs * (€/kg Live Weight)	Income/Sow/ Year (€)	Change (%)
2014	19.78	111.82	1.25	2765	**+30**
2021	25.70	118.10	1.25	3592	

* Source: https://ec.europa.eu/eurostat/databrowser/view/tec00033/default/table?lang=en (accessed on 1 May 2023) [19,20]. Note: 2022 data are not available yet.

## Data Availability

Detailed data, pictures and notes are available via the corresponding author’s e-mail address.
